# Perspectives on using integrated tick management to control *Rhipicephalus microplus* in a tropical region of Mexico

**DOI:** 10.3389/fvets.2024.1497840

**Published:** 2024-11-22

**Authors:** Rodolfo Lagunes-Quintanilla, Ninnet Gómez-Romero, Nancy Mendoza-Martínez, Edgar Castro-Saines, Dulce Galván-Arellano, Francisco Javier Basurto-Alcantara

**Affiliations:** ^1^Centro Nacional de Investigación Disciplinaria en Salud Animal e Inocuidad—INIFAP, Carretera Federal Cuernavaca—Cuautla, Jiutepec, Mexico; ^2^Vaccinology Laboratory, Department of Microbiology and Immunology, Facultad de Medicina Veterinaria y Zootecnia, Universidad Nacional Autónoma de México, Mexico City, Mexico; ^3^Posgrado en Ciencias de la Producción y de la Salud Animal, Facultad de Medicina Veterinaria y Zootecnia, Universidad Nacional Autónoma de México, Mexico City, Mexico; ^4^Centro de Investigación y Estudios Avanzados en Salud Animal, Facultad de Medicina Veterinaria y Zootecnia, Universidad Autónoma del Estado de México, Toluca de Lerdo, Mexico

**Keywords:** cattle, Integrated Tick Management, *Rhipicephalus microplus*, Mexico, Veracruz

## Abstract

The *Rhipicephalus microplus* tick is widely recognized as the most economically significant ectoparasite affecting cattle globally, particularly in the Neotropical region. In Mexico, at least 65% of the cattle are infested with *R. microplus* and are susceptible to tick-borne diseases. Integrated tick management strategies are required to maintain compatible levels of animal production and reduce the reliance on chemical acaricides for tick control. Therefore, this paper aims to analyze current methods for controlling tick infestation in extensively raised cattle using Integrated Tick Management (ITM) and to propose an ITM program suitable for implementation in the humid tropical region of Veracruz, Mexico.

## 1 Introduction

The ticks are obligatory hematophagous ectoparasites of vertebrate hosts and are vectors and reservoirs of several microorganisms, such as protozoa, bacteria, and viruses (1). The tick *Rhipicephalus microplus* in livestock production is cattle’s most economically significant ectoparasite worldwide since it can significantly decrease live-weight gain, milk and meat production, and leather quality in the Neotropical region. These effects are caused by tick infestation and potentially pathogen transmission, such as *Babesia bigemina*, *B. bovis*, and *Anaplasma marginale* (2, 3). Moreover, climate change has contributed to the expanded range of ticks, changing the distribution, dynamics, prevalence, and seasonal activity patterns, resulting in a high tick population active throughout the year. This phenomenon occurs mainly in Africa, Australia, and Latin America (4, 5).

The cattle industry is one of Mexico’s most important agricultural activities, producing 36.3 million heads of dairy and beef cattle (6). The exportation of live cattle from Mexico to the USA represents annual earnings estimated at USD 700 million; for example, around 1.2 million live cattle were exported in 2023 (7). In this context, maintaining adequate sanitary control is crucial to reduce tick populations. However, most animals are located in the Mexican tropics, where at least 23 million beef are exposed to tick infestations because of the humid tropical climate and conditions suitable for *R. microplus* development. This issue has been supervised by the National Service of Agro-Alimentary Public Health Safety and Quality (SENASICA) through the official national campaign against *R. microplus*, determining the zoo-sanitary measures applicable to protect tick-free areas in Mexico. According to official reports, 65% of the cattle in Mexican territory are infested with *R. microplus* and are susceptible to tick-borne diseases (8). Consequently, national economic losses have been calculated at USD 573.6 million annually (9).

Currently, the common method to control tick infestations in Mexico is using acaricide products from different chemical families. Nonetheless, inadequate and excessive treatments have serious drawbacks associated with the appearance of multi-resistant tick strains, chemical residues in food products (meat and milk), adverse environmental impacts, and high production costs for ranchers (3, 10). These aspects emphasize the need for alternative methods, such as producing or introducing Zebu cattle (*Bos indicus*), pasture and paddock management, use of plant extracts, microbial control (bacterial, nematode, and fungal), and vaccination (11–15). Most of these practices are wanted to reduce the field’s tick population. However, not all of them have the desirable efficacy when applied individually. For this reason, it is necessary to design and implement an Integrated Tick Management (ITM) program according to each geographical region in Mexico. This strategy involves combining several control methods that are accessible to use, environmentally friendly, and less likely to develop resistance. Therefore, the aim of this paper is (1) to discuss the current situation regarding the control methods used to reduce tick infestation in cattle raised in extensive systems based on a review of research addressing the implementation of ITM in the Neotropical region and (2) to provide a proposed ITM program design that can be implemented in a humid tropical region of Veracruz, Mexico.

## 2 Current status

In recent years, the national beef and milk production industry has increased significantly, producing 2 million tons of beef and veal, 13 million tons of milk, and 300,000 tons of beef exports (16, 17). The production system in the country is based on the cow-calf under three systems: purebred, multiplier, and dual-purpose. The first two are developed in states in Northern Mexico and are characterized by registered animals, superior genetics, and intensive milk and meat production farms. The dual-purpose systems are applied in 70% of the total cattle farmers from the tropical and subtropical regions of Mexico. Family farms have traditionally preferred this system due to its flexibility to produce milk and meat (18). However, cattle are largely affected by tick infestations, demanding continuous efforts to manage tick populations through several control methods.

Currently, control of tick infestation strategies in Mexico is based on an acaricide application. Amidines, synthetics pyrethroids (SP), organophosphates (OP), macrocyclic lactones (ML’s), phenylpyrazolones, and fluazuron (10, 19) are the main types of molecules commercially distributed in the country (9, 15). In addition, cattle tick infestations are controlled using microbial and plant extract control approaches and vaccination with limited application due to their implementation being based on the knowledge of cattle producers.

### 2.1 Chemical control

Acaricides are the main method of control for *R. microplus* globally. Most acaricides and endectocides can act directly on the nervous system and exert their effect systemically either after absorption by the host tissues or by direct contact with the ectoparasites after external application through different mechanisms such as inhibitors of acetylcholinesterase, sodium channel blockers, and voltage-dependent modulators, glutamate-gated chloride (GluCl) channel modulators, inhibitors of chitin biosynthesis, GABA-gated chloride channel blockers, mitochondrial complex electron transport inhibitors and uncouplers of oxidative phosphorylation (20).

These chemical treatments have contributed to reducing the field population of ticks and improving cattle productivity; however, their inadequate and excessive use has resulted in the selection of chemical-resistant tick strains and, in some regions of the world, multi-resistant strains (21). Recently, in Mexico, the *R. microplus* populations have developed resistance to multiple classes of acaricides, particularly in the Tamaulipas, Veracruz, and Yucatan states, making the tick control inefficient and increasing the costs associated with the treatments to cattle producers (10, 22–25). This situation emphasizes the need to implement quick strategies to reduce the *R. microplus* population. Hence, avoid relying solely on one tactic method for tick control. Conversely, combining multiple approaches and minimizing the risks related to food and environmental contamination is essential. This includes preventing the establishment and spreading of multi-resistant ticks into free zones and restrictions on cattle export (26, 27).

### 2.2 Cattle breeds

It is well known that Zebu (*Bos indicus*) cattle are more resistant to *R. microplus* infestation than European (*B. taurus*) breeds, suggesting that it is due to an enhanced T cell-mediated immune response; studies have demonstrated a significant reduction in the response of lymphocytes on *B. taurus* causing immunosuppressive effects after infestation of *R. microplus* (28). In addition, several observations in Zebu cattle managed in naturally infested pastures indicated significantly higher levels of tick saliva-specific IgG1, IgG2, and IgE antibodies than Holstein cattle, suggesting that Zebu breeds recognized more tick salivary proteins and, therefore, presented lower tick loads (29, 30). In general, the level of tick infestation is influenced by the degree of susceptibility of cattle, grazing, and region. Pure or crossbred Zebu cattle are more adaptable to tropical and subtropical areas and more resistant to *R. microplus* and other tick species (31). However, this cattle breed is not well accepted due to its low productivity in the cattle industry.

### 2.3 Pasture and paddock management

The paddock strategies for reducing the impact of tick infestation are concerned with applying ITM. Among the strategies for paddock management are selective grazing and pasture rotation. Selective grazing aims to manipulate the tick microenvironment by using grasses or legumes with repellent properties to prevent tick larvae from climbing the stem and reaching the leaves, hence dying of starvation, dehydration, and asphyxiation (32). The grasses such as *Mellinis minutiflora*, *Brachiaria brizantha,* and *Andropogon gayanus* are popularly known in tropical and subtropical regions, characterized by secreting secondary metabolites expressed as viscous fluid of characteristic odor. This oily material is reportedly responsible for molasses grass’s ability to reduce cattle tick infestation by repelling or killing tick larvae (33, 34).

The pasture rotation is based on dividing an area into paddocks with frequent and scheduled movement of cattle, providing rest periods between paddocks (from one to another) to allow the recovery and growth of grass. Therefore, it delays cattle contact with tick larvae, interfering with the life cycle. This results in the mortality of viable larvae populations due to starvation or dehydration (35). Thus, it helps to optimize forage resources within the paddocks and reduces parasite–host interaction (36). Additionally, several studies have established the necessary rest periods for paddocks to decrease the number of viable larvae in the environment (35–37). Recently, in a study performed in a tropical region of Veracruz, Mexico, the cattle presented fewer larvae when using a 45-day rest period in the paddocks, suggesting that this interval reduces the percentage of larval viability in the paddocks (12). It is important to note that several factors can influence the use of tick control, such as the dynamics of tick population, climate, pasture, number and divisions of paddocks, and number of animals to be used (36).

### 2.4 Plants as tick repellents/acaricides

Plant extracts have been researched for use in tick control as a potentially environmentally friendly alternative with fewer negative consequences to cattle. Recently, emphasis has been placed on searching for and identifying plants (crude extracts, essential oils, and secondary metabolites) with repellent and acaricidal properties against the population of *R. microplus* (13). The plant species with the highest repellent/acaricide effect reported are *Lamiaceae*, *Asteraceae*, *Rutaceae*, *Fabaceae*, *Solanaceae*, *Meliaceae*, *Poaceae*, *Euphorbiaceae*, and *Piperaceae* (38–40). In addition, *in vitro* assays showed variable efficacies ranging from 37–100% against several life cycle stages of tick species (egg, larvae, nymphs, and adults). These results demonstrated that terpenoids are the most secondary metabolites identified with such activity (13, 38–40). However, some limitations have been detected in evaluating plants as a tick repellent, such as the efficacy of plant extract has been reduced when tested in field trials, most plant products do not persist in the environment, possibly by temperature or degradation, and the cultivation/collection of plants depends on the climatic condition. Also, the chemical composition may vary depending on the climate; consequently, different efficacy results have been observed (13).

### 2.5 Microbial control

This method uses natural enemies and is widely documented for pest management and control. Entomopathogenic fungi, such as *Metarhizium anisopliae*, have been described as a potential biocontroller of ticks (41, 42). The fungus mechanism of action in ticks consists of cuticle penetration; once attached, the fungus forms an appressorium organ and then breaks down the cuticle through mechanical pressure until it reaches the hemocoel. The fungus develops and releases blastospores that colonize several tick organs, resulting in death. Subsequently, the hyphae emerge and form conidia, which are released into the environment (43). The efficacy of *M. anisopliae* has been validated in many laboratory trials against larvae and adult *R. microplus* ticks, considering the optimal temperature and relative humidity for this class of fungus, ranging from 25°C and 75%, respectively (42, 43).

Additionally, the fungus has been successfully deployed in pastures with high tick infestation and has also been used as an external ixodicide for infested cattle (14). The formulation application of conidia with wheat bran (2 × 10^9^ CFU/m^2^) using an electric sprayer on infested tick pasture resulted in a significant reduction in tick larvae, 94 and 58% at 14- and 21 days post-application, respectively (44). Other studies demonstrated the efficacy of *M. anisopliae* using a solution containing 1×10^8^ conidia/mL at 15-day intervals, reducing 40 and 91% of the number of ticks in the cattle naturally infested (45). Recently, a commercial formulation of *M. anisopliae* has been developed for pest control in agriculture; this product demonstrated an efficacy of around 75% in tick field trials. The application involved spraying the formulation all over the body of cattle. Each animal was sprayed with 4 L of the formulation with two treatments applied in 3-day intervals (46). The development of products based on fungi for controlling pests in agriculture, including insects and ticks, has increased significantly worldwide. However, differences between results have been reported, possibly due to environmental conditions, fungi isolates used, the distinct tick population, and the methodology used for efficacy evaluation (46, 47).

### 2.6 Anti-tick vaccines

Vaccines represent a promising alternative for controlling *R. microplus* because it has been possible to reduce tick infestation while blocking the transmission to their hosts. Additionally, it is a friendly and sustainable method because it does not contaminate the environment or animal products and avoids any risk to animal or human health (48). In this regard, tick vaccines have been characterized by identifying proteins categorized as concealed or non-concealed antigens. However, few of these antigens have been evaluated in field immunization trials (49). The recombinant Bm86 antigen is the only commercially available in Latin America and Mexico, with efficacy between 51 and 100% in controlled infestations with *R. microplus* and *R. annulatus* (50). Briefly, anti-tick vaccines stimulate the humoral immune response of cattle through the production of anti-Bm86 protective antibodies in the following manner: when cattle are inoculated with the initial dose containing the Bm86 antigen, the antigen-presenting cells (APCs), such as dendritic cells (DCs), macrophages (Macs), and in some circumstances, B cells, process and present the antigen in the lymph node via the Bovine major histocompatibility complex (BoLA). Then, activated dendritic cells interact with naive T cells, differentiating them into effector T-helper (CD4+) cells, which would stimulate via Th2 through cytokines (IL-4, IL-5, and IL-13) and interactions CD40/CD40L the activation of the B cells, forming memory B cells and plasma cells in the lymphoid germinal centers (51). Subsequently, the plasma cells migrate to the bone marrow and induce the production of antigen-specific antibodies (IgG anti-Bm86), while the memory B cells located in the spleen, blood, lymphoid organs, and barrier tissues are waiting for a booster dose of the antigen. The anti-IgG’s bind to the Bm86 antigen and activate the complement system (classical pathway), resulting in the lysis of enterocytes, disrupting their function and causing a decrease in reproductive tick’s parameters, thereby affecting progeny (52, 53). However, commercial tick vaccines have limitations due to sequence variations in the target protein among different tick strains in America, including Mexico (54, 55). For this reason, new strategies for discovering tick vaccines have recently been sought, pretending to develop regional vaccines that could improve the efficacies against various geographical strains of *R. microplus* (49, 50).

## 3 Future perspective and challenges

### 3.1 Integrated control strategies for *Rhipicephalus microplus* ticks in tropical regions

The control of *R. microplus* infestations mainly involves using chemical acaricides. However, in some parts of the Neotropical region, farmers have been implementing ITM strategies combining two or more methods/technologies to manage *R. microplus*, intending to maintain compatible levels of animal production and avoid dependency on a single strategy to control tick infestations (26). Some shreds of evidence are shown in Table 1. Employing other methods in addition to acaricide control, such as essential oils (EOs) and compounds found in essential oils (CEOs) with conventional acaricides, demonstrated that thymol increased the efficacy of amitraz around 74% against engorged females *R. microplus* under field conditions in Brazil (56). Also, combining entomopathogenic fungi and commercial acaricides (SP, OP) achieved high efficacy, reaching 97.9% against tick-resistant populations in field conditions (57). The anti-tick vaccine is the most common technology that combines with conventional acaricides to manage cattle tick infestations. The integrated use of recombinant Bm86 vaccine reported a reduction of chemical acaricides between 50 to 80% over a 6-month period in cattle naturally infested in Puerto Rico; additionally, it decreased the incidence of tick-borne diseases (58). In the same context, vaccination with Bm86 in combination with ML’s reduced the number of larvae by 81% and the fertility index for four months, providing significant and prolonged control compared to the methods applied separately (59). Studies in Cuba and Venezuela have combined tick vaccines with chemical acaricides to control *R. microplus*, resulting in fewer acaricide treatments in cattle, ranging from 68 to 87% (60, 61). In Mexico, tick vaccines were combined with an amidine to control *R. microplus,* and the annual number of acaricide applications was reduced from 24 to 7 in 9-year period (62, 63). These reports indicate that the association of chemical acaricides with the EOs, CEOs, biological agents, and tick vaccines can be used in ITM to ensure effective and sustainable practices (26).

**Table 1:** Integrated strategies in controlling *Rhipicephalus microplus* tick infestations in tropical regions.

Strategy	Evaluation	Stage/Tick	Efficacy	Citation
Amitraz + Thymol (Thymol monoterpenes and Thymol acetates)	Field conditions	engorged females	74%	(56)
*M. anisopliae +* SP + OP (Cypermethrin + chlorpyrifos)	Field conditions	all life stages (acaricide-resistant strain)	97.90%	(57)
Vaccine + acaricides	Field conditions	all life stages	50–80% (reduction in acaricides treatments)	(58)
Vaccine + ML’s (Moxidectin 1%)	Field conditions	larvae	81%	(59)
Vaccine + acaricides (amidines, SP, OP)	Field conditions	all life stages	87% (reduction in acaricides treatments)	(60)
Vaccine + acaricides	Field conditions	all life stages	83%	(61)
Vaccine + amidines	Field conditions	all life stages (acaricide-resistant strain)	near 100%	(63)

### 3.2 Perspective using ITM practices in a tropical region of Veracruz, Mexico

Veracruz State has a cattle population 6,112,220, representing 16.6% of the national herd (64). It is the leading beef producer in Mexico, contributing 13% of the total national production with 287,065 tonnes of beef (65) and 807,075 liters/year of milk production (66). In the State, the main cattle production system is dual-purpose, traditionally preferred in the tropics due to producing meat and milk at low cost using Zebu (mainly Brahman, Gyr, and Guzerat) and *B. taurus* x *B. indicus* breeds, adaptability, less investment requirements, and the use of extensive grazing as the main food source (18). Veracruz plays a crucial role in the movement of cattle exportation from Mexico to the USA. However, this transit has increased the spreading of the *R. microplus* tick population resistant to the principal acaricide families (amidines, SP, OP, ML’s) (19, 22–24, 67).

For ITM to be adequate and effective, it is important to consider the environmental conditions of each region, the population dynamics of the ticks to be controlled (tick species), and the emergence of populations resistant or multi-resistant to acaricides. The following proposal for ITM is designed for a humid tropical region in Veracruz, Mexico.

The location is characterized by a humid tropical climate with three established seasons: rainy (June–September), winter (October–January), and dry season (February–May). According to the Meteorological Service database, the average annual temperature is 25.5°C with a relative humidity between 82–90% and an average rainfall of 1,387 mm per year (68). The population dynamics of the *R. microplus* ticks have been reported in this zone (12, 69), with infestations occurring throughout the year and representing approximately 80% of the health issues affecting cattle in this region (70). However, the highest tick infestations are from June to October (mainly coinciding with the rainy season), resulting in 4–5 generations annually (12, 69). According to these data, it was possible to classify the risk of cattle tick infestation as low (winter season), medium (dry season), and high (rainy season).

**Figure 1:**
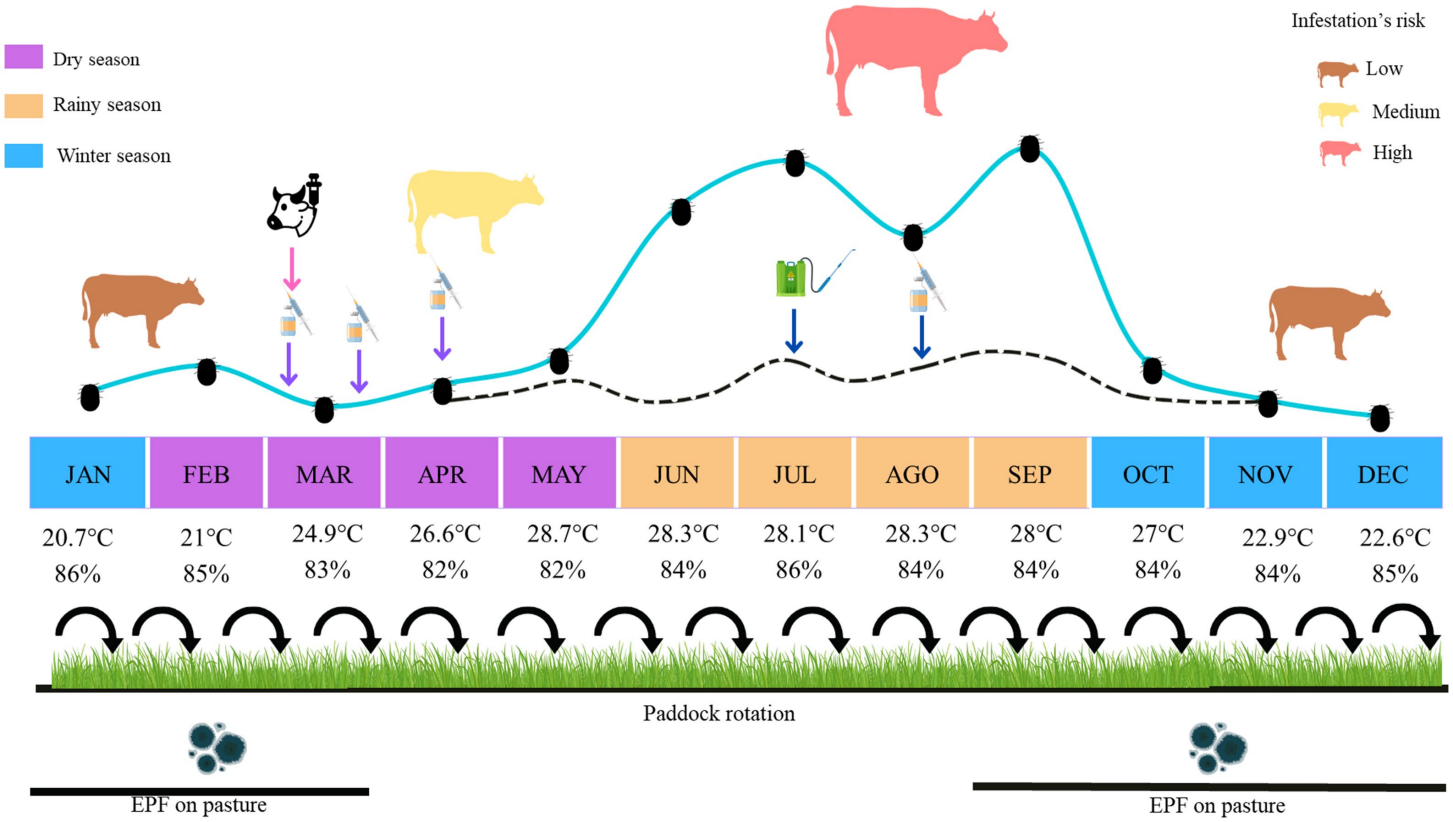
Graphic representation of the integrated tick (*R. microplus*) control proposal in a humid tropical region of Veracruz, Mexico. The solid line represents the levels of cattle tick infestation, characterized by four distinct peaks occurring in February, June, July, and September, as per the population dynamics observed in the state of Veracruz. The dotted line represents the target infestation levels to keep *R. microplus* populations at controllable levels. The winter months are marked in blue, the dry season in purple, and the rainy season in brown; below each month are the average temperature (°C) and relative humidity (%) recorded in the Mexican humid tropics. The categorization of cattle by risk of tick infestation is indicated, with brown, yellow, and red representing low, medium, and high risk, respectively. The application of the vaccination scheme is denoted by purple arrows, the administration of ML’s long-acting by a pink arrow, and the use of a second acaricide (e.g., by spraying) during the rainy season by a navy-blue arrow. Additionally, the proposal includes paddock rotation throughout the year, with a 45-day rest period. Furthermore, the application of entomopathogenic fungi (EPF) on pastures is recommended during the months of January to March and September to December.

The proposed program for the integrated control of *R. microplus* in the geographical area described is shown in Figure 1. In this program, two strategies are proposed to decrease tick infestations: reducing the number of ticks available in the paddocks and the number of ticks on the cattle. Firstly, the use of a pasture rotation with 45 days of rest periods between paddocks (adapted to vegetation, availability, and number of paddocks) (12), and application of entomopathogenic fungi (*M. anisopliae*) on infested tick pasture every 14 days from January to March and September to December to covering the seasons with highest tick infestations. The treatments consist of 1 × 10^10^ conidia/m^2^ added to 45 g of wheat bran and adjusted to a dose of 50 kg/ha by manual scattering (44, 71). The treatments should be in the late afternoon (6–7 p.m.) to avoid the effects of sunlight and UV radiation. In second place, ticks will be collected to assess the susceptibility to acaricides through the larval packet test (LPT) and the larval immersion test (LIT) to determine the degree of resistance (72) and propose a desirable acaricide. Afterward, the application of ML’s long-acting such as ivermectin 3.15% administrated to cattle at 630–700 μg/kg dose or moxidectin 1% at 200 μg/kg dose in a single subcutaneous injection under the loose skin located behind the shoulder, according to label instructions (59, 73). This application is proposed in March and is combined with a 3-dose vaccination scheme (days 0, 30, 50). Each 2 mL vaccine dose included 100 μg of recombinant Bm86 or Subolesin antigen immunized subcutaneously in the neck using a 5 mL syringe and an 18 G needle (49, 74). This scheme is intended to reduce tick population during the dry season and to stimulate the production of protective IgG antibodies in cattle. These cattle population will be challenged during the rainy season when the tick infestation is considerably high. Likewise, during the rainy season, a second acaricide should be applied, such as amitraz 12.5%, administrated at 250 μg/mL in July to reduce the number of ticks. Each animal will be sprayed with 5 L of emulsion uniformly on the whole body (56). For treatment, cattle will be contained in a comprehension ramp to facilitate the correct application using a backpack sprayer. In August, the vaccine booster will be performed to generate a secondary and specific response that triggers high levels of protective IgG antibodies to reduce tick populations on cattle, which usually increase during September.

Finally, this ITM aims to reduce ticks in successive generations, reduce the number of acaricide treatments per year, implement targeted acaricide treatments when the number of engorged *R. microplus* ticks per animal is greater than ~40, delay the emergence of tick-resistant populations, maintain compatible levels of animal production, and create enzootic stability for tick-borne diseases.

## 4 Challenges

Farmers in tropical regions should consider implementing ITM’s to control cattle ticks. ITM’s represent the most effective strategy for establishing sustainable and successful tick population management (26, 27, 36, 51, 57–63). However, various factors, including those mentioned above, hinder its adoption in Mexico. Moreover, other gaps require special attention, such as the promotion of good practices (rational and responsible use of acaricides), implementation of ITM’s regionally rather than individually, the limited control of ticks in wildlife which fosters the expansion of tick populations, and the impact of climate change and microclimates that facilitate the proliferation and propagation of tick populations. Furthermore, ITM’s success should be considered by the participation of government institutions, such as SENASICA, the veterinary pharmaceutical industry, and regional and local rancher associations (26, 27). They must be involved in the process of technical assistance, training, resources, and implementing recommendations to control tick populations for specific regions. Also, information on population dynamics, tick species, and the resistance situation should be distributed by region or locality. This information must be critical for enhancing tick control management, especially concerning practices that mitigate acaricide resistance, raising farmers’ awareness of the situation, and selecting an effective control method. In addition, the willingness, motivation, and knowledge of farmers are crucial in the Mexican cattle industry. A significant part of the livestock farming in the country is carried out by small producers who may not have access to information or follow outdated control practices, which could hinder tick control. To address this challenge, it is important to focus on reaching out to small farmers, sharing knowledge, and providing technical support to implement ITM’s effectively. Finally, these perspectives highlight the importance of involving multiple sectors to implement integrated approaches, making a cost-effective and sustainable cattle production in Mexico.

## Data Availability

The original contributions presented in the study are included in the article/supplementary material, further inquiries can be directed to the corresponding author.
